# Information and Communications Technologies Enabling Integrated Primary Care for Patients With Complex Care Needs: Scoping Review

**DOI:** 10.2196/44035

**Published:** 2023-04-19

**Authors:** Farah Tahsin, Alana Armas, Apery Kirakalaprathapan, Mudathira Kadu, Jasvinei Sritharan, Carolyn Steele Gray

**Affiliations:** 1 Institute of Health Policy, Management and Evaluation University of Toronto Toronto, ON Canada; 2 Bridgepoint Collaboratory for Research and Innovation Lunenfeld-Tanenbaum Research Institute Sinai Health Toronto, ON Canada; 3 Department is School of Public Health Sciences Faculty of Health Sciences The University of Waterloo Waterloo, ON Canada

**Keywords:** information and communications technology, multimorbidity, integrated care, primary care, review method, scoping, complex care, mobile phone

## Abstract

**Background:**

Information and communications technologies (ICTs) are recognized as critical enablers of integrated primary care to support patients with multiple chronic conditions. Although ICT-enabled integrated primary care holds promise in supporting patients with complex care needs through team-based and continued care, critical implementation factors regarding what ICTs are available and how they enable this model are yet to be mapped in the literature.

**Objective:**

This scoping review addressed the current knowledge gap by answering the following research question: What ICTs are used in delivering integrated primary care to patients with complex care needs?

**Methods:**

The Arksey and O’Malley method enhanced by the work by Levac et al was used to guide this scoping review. In total, 4 electronic medical databases were accessed—MEDLINE, Embase, CINAHL, and PsycINFO—collecting studies published between January 2000 and December 2021. Identified peer-reviewed articles were screened. Relevant studies were charted, collated, and analyzed using the Rainbow Model of Integrated Care and the eHealth Enhanced Chronic Care Model.

**Results:**

A total of 52,216 articles were identified, of which 31 (0.06%) met the review’s eligibility criteria. In the current literature, ICTs are used to serve the following functions in the integrated primary care setting: information sharing, self-management support, clinical decision-making, and remote service delivery. Integration efforts are supported by ICTs by promoting teamwork and coordinating clinical services across teams and organizations. Patient, provider, organizational, and technological implementation factors are considered important for ICT-based interventions in the integrated primary care setting.

**Conclusions:**

ICTs play a critical role in enabling clinical and professional integration in the primary care setting to meet the health system–related needs of patients with complex care needs. Future research is needed to explore how to integrate technologies at an organizational and system level to create a health system that is well prepared to optimize technologies to support patients with complex care needs.

## Introduction

### Background

There is a growing number of patients living with 2 or more chronic conditions in Canada and worldwide [1,2]. These individuals not only manage multiple chronic conditions [1] but also experience additional biopsychosocial challenges [3,4]. Living with multiple chronic conditions can introduce additional complexity into this patient population’s care management experience, such as high symptom and treatment burden [5,6]. In addition, this patient population can have complicated and contradictory treatments or medication regimens [7] that require care from multiple health professionals and services spanning various health and social care settings [8]. Owing to the multitude of challenges this patient population faces in the current disease-specific care model [9], this patient population is often referred to as patients with complex care needs or complex patients. Navigating a fragmented care system can leave patients with complex care needs feeling overwhelmed and dissatisfied with their care [10,11]. An agile and coordinated care system can better address this population’s challenges.

Integrated care models that involve primary care are well positioned to support people living with chronic conditions as most chronic disease–related care is delivered in this setting [12]. The definition of integrated care is a coordinated, collaborative, multidisciplinary, and person-centered care delivery system [13]. In recent years, integrated care approaches have been shown to support improved care coordination within primary care [14,15]. However, implementing integrated primary care models is an ongoing challenge within health systems.

The Rainbow Model of Integrated Care (RMIC) offers a conceptual way of understanding integrated care, suggesting that integration can occur at different levels (clinical, professional, organizational, and system) and is enabled through functional and normative mechanisms [13]. This validated model is helpful in understanding the different types and degrees of integration that a care model or intervention aims to achieve [16,17]. Each level of integration (clinical, professional, organizational, and system) may require different strategies, tools, and mechanisms to support integration efforts, such as care coordination across health professionals for effective care continuity and coordination of services [18]. Information and communications technologies (ICTs) can enable these types of integration efforts regarding care continuity or shared decision-making by offering their functionalities, such as sharing patient data across professionals and organizations [19,20]. In the health care setting, ICT is used as an umbrella term that includes different types of health technologies [21]. Therefore, to operationalize this term for this study, we have used the World Health Organization ICT definition in the context of health systems, which is as follows: health services and information delivered through the combined use of the internet and other electronic communication technologies [22,23]. ICT-enabled integrated care can be helpful for patients with complex care needs in terms of care coordination and as a decision support tool as this patient population may require multiple support services from multiple health professionals and organizations [18].

Textbox 1 shows the RMIC levels and how ICTs can support each component.

**The Rainbow Model of Integrated Care levels and examples of information and communications technologies (ICTs) that can support integration activities in the primary care model.**

*Clinical level*

- Refers to the coordination of person-centered patient care across multiple conditions, care teams, settings, and time to provide efficient care
- Example of ICTs: patient portal to access information across services [24] and telehealth to deliver coordinated services remotely [25]

*Professional level*

- A means to promote teamwork and collaboration among multidisciplinary teams
- Example of ICTs: videoconferencing among providers [26] and access to patient data across providers [27]

*Organizational level*

- Interorganizational care coordination, including common administrative mechanisms to deliver comprehensive services to a defined population
- Example of ICTs: shared patient electronic medical records among primary care and community services [28]

*System level*

- An alignment of rules, regulations, and policies within a system
- Example of ICTs: telehealth reimbursement policies for primary care providers [29]

*Formative level*

- Key functions to support care integration
- Example of ICTs: the care model being situated in an integrated network where financial, information, and management systems are coordinated [30]

*Normative level*

- Having a common frame of reference, shared values, and goals for service delivery between organizations, stakeholders, and providers
- Example of ICTs: a shared goal of accelerating internet-based care adoption during COVID-19 [31]

Another way to conceptualize how technology can support patients with chronic conditions is the eHealth Enhanced Chronic Care Model (eCCM). This model builds on the foundational Chronic Care Model introduced by Wagner [32] that has been used to guide chronic disease management for patients with complex care needs in primary care and other settings [33]. The eCCM version offers a salient way to identify how ICTs and ICT-generated information can aid in improving patients’ health outcomes by increasing patients’ and providers’ knowledge of chronic disease management [21]. Textbox 2 shows the key components of the eCCM.

**Key elements of the eHealth Enhanced Chronic Care Model.**

*eCommunity resources*

- Refers to developing strategies that link with community organizations and virtual health-related communities
- Example of information and communications technologies (ICTs): health-related social networks and web-based communities that facilitate care connections

*Health system enhancements*

- Refers to the strategies in place to support patient engagement and self-management support
- Example of ICTs: web-based health platforms and mobile health that support quality improvement

*Delivery system design enhancements*

- Refers to technologies that facilitate teamwork practice to deliver care efficiently
- Example of ICTs: electronic health records (EHRs) and web-based health platforms that facilitate information sharing

*Self-management support enhancements*

- Refers to the patient’s active role in managing their care through using technology
- Example of ICTs: health apps and web-based resources that support patients’ self-management skills

*Clinical decision support enhancements*

- A means for providers and patients to have access to evidence-based clinical guidelines, protocols, care standards, and self-management resources to make an informed decision
- Example of ICTs: web-based platforms and EHRs to access protocols and guidelines on the internet

*Clinical information system enhancements*

- Management of information systems (ie, patient databases and patient portals or personal health records) to facilitate efficient care
- Example of ICTs: mobile health apps and web-based platforms to coordinate care and monitor patients’ health status

### Objectives

Taken together, the RMIC and eCCM offer useful suggestions on how ICT may be of use to enable integrated primary care delivery for patients with complex care needs. Although these frameworks and emerging literature in the field of digital health and care integration provide some evidence of how technology may be of service to patients with complex care needs, there has not been a comprehensive systematic review of the literature focused on how ICT supports integrated primary care models to identify current trends or gaps in research. Therefore, the purpose of this knowledge synthesis was to scope the current literature on the types of ICTs that are being used to support patients with complex conditions in integrated primary care settings while also identifying potential areas for continued research.

## Methods

### Overview

A scoping review approach was used to systematically map and identify relevant literature at the crossroads of integrated primary care models, ICTs, and patients with complex conditions. Scoping reviews capture a breadth of literature, which can effectively identify emerging literature as well as evidence gaps on a topic of interest [34]. Given that ICT-based integrated primary care is comparatively a newer model of care, a scoping review is an ideal approach for this study. To conduct a rigorous review, we followed the six stages of the scoping review framework by Arksey and O’Malley [34] enhanced by the work by Levac et al [35]: (1) identifying the research question; (2) identifying relevant literature; (3) selecting the studies; (4) charting the data; (5) collating, summarizing, and reporting the articles; and (6) disseminating knowledge. Each of these 6 stages is described in detail in the following sections. For further details, the study protocol has been published elsewhere [36].

### Stage 1: Identifying the Research Question

The guiding research question for this scoping review was as follows: What ICTs are used in the delivery of integrated primary care to patients with complex care needs? The subquestions were as follows: (1) Which technologies are being used for this patient population? What are the functionalities and characteristics of these technologies? (2) How are these technologies being used in the integrated primary care model in terms of care integration? (3) What implementation factors are being reported in the selected studies that describe ICT-enabled integrated primary care models?

These questions were selected as there has been no systematic documentation in the current literature of *what* sort of ICTs are available to support an integrated primary care model or *how* ICTs are enabling the process of care integration in primary care settings. In addition, the third subquestion was posed given the importance of implementation strategies in the successful use of ICT-based interventions in the integrated primary care model [37]. Given the interconnectedness of the primary research questions and the 3 subquestions, a single search strategy and review were determined to be sufficient, and addressing all 3 questions in a single study was expected to lead to a richer and more meaningful analysis and findings.

### Stage 2: Identifying Relevant Literature

To operationalize the research questions, we identified and defined 4 major concepts to inform our search strategy. The 4 concepts and their key definitions are outlined in Textbox 3. Multimedia Appendix 1 shows the keywords associated with each concept for each category.

Relevant studies were identified by searching the following electronic databases of published literature: Ovid MEDLINE, Ovid Embase, EBSCO CINAHL, and Ovid PsycINFO. The MEDLINE search strategy was developed first and peer reviewed using the Peer Review of Electronic Search Strategies tool. This search strategy was then translated to the remaining 3 databases. The searches were limited to articles published between January 2000 and December 2021. Articles published before 2000 were excluded as a preliminary search showed that there were very few articles published on ICTs during this time. The MEDLINE search strategy was included in the published protocol [36]. In addition, the search strategy for PsycINFO can be found in Multimedia Appendix 2. The initial search of all databases was completed in the summer of 2019 and then updated in December 2020 and again in December 2021.

**Key definitions.**

Information and communications technologies (ICTs) [23,38]

- Health care–related services and information are delivered through the combined use of the internet and other electronic ICTs. In this study, the eHealth Enhanced Chronic Care Model is used to categorize ICTs based on the primary functions of a technology (ie, decision support).

Integrated care model [13]

- This refers to a coordinated collaborative, multidisciplinary, and person-centered care delivery system.

Patients with complex care needs [9]

- Individuals with multiple chronic conditions often encounter additional psychosocial challenges. The complexity of their conditions affects treatment, health outcomes, and quality of life.

Primary health care [12]

- This refers to the first point of contact to health care that provides comprehensive community-based service. Primary health care is most often delivered by general practitioners or family physicians.

### Stage 3: Study Selection

The search results were imported into the knowledge synthesis software Covidence. The inclusion and exclusion criteria were imported into the software as well. The inclusion and exclusion criteria were decided a priori and were used for screening citations first, for the title and abstract review, and then again for the full-text review. Both levels of review were conducted in duplicate by 2 reviewers.

To be included in the review, articles were required to report on an intervention that (1) had an ICT-enabled health care model, (2) was based on an integrated health care model or team-based care, (3) included adult patients with complex care needs, and (4) took place in a model that included primary care. If the intervention had a target population of (1) individuals aged <18 years (given the focus of this review on adults with complex care needs) or individuals with (2) cancer or (3) mental disorders, it was excluded as cancer care and mental health provision have unique care pathways that may not be translatable into integrated primary care settings [39,40]. Any published article, including quantitative studies, qualitative studies, mixed or multimethods research, both comparative (eg, randomized, controlled, cohort, or quasi-experimental) and noncomparative (eg, survey and narrative audit) methods, educational materials, and reports, could be included in the review if it met the aforementioned inclusion criteria. These inclusion and exclusion criteria were published in the study protocol [36].

To ensure consistency between reviewers, a series of training exercises and discussions were held before commencing title and abstract screening. At first, all 6 members of the research team screened a random sample of 30 articles to assess interrater agreement. Interrater agreement for study inclusion was calculated using percentage of agreement; when it reached >70% across the team, we proceeded to the next stage. If a lower agreement was observed, the team had a discussion about eligibility criteria, and another pilot test was conducted. In total, 6 rounds of pilot tests were required for the title and abstract screening of a random sample of a total of 110 citations. After the initial agreement between the 6 screeners was established, they were divided into groups of 2 for an efficient screening process. The pairs worked together to screen titles and abstracts for inclusion. Any disagreements within pairs were resolved through weekly group discussions.

For the full-text screening stage, 3 rounds of pilot tests were conducted on a random sample of 30 articles. All 6 team members participated in the pilot-testing round. In each round, interrater agreement was calculated using percentage of agreement. In the third round, we reached >70% consensus across the team. Subsequently, the 6 team members were divided into 3 groups consisting of 2 group members each. All 3 pairs screened the full texts of potentially relevant articles to determine inclusion using the inclusion and exclusion criteria. All discrepancies were resolved through weekly group discussions.

### Stage 4: Data Extraction

All the included studies were reviewed and charted independently by 6 team members. To ensure consistency between team members regarding data extraction, the entire team pilot-tested the data abstraction form on 3 articles that were randomly selected. Full data abstraction began only after full agreement was reached. The full agreement was decided based on percentage of agreement, and it reached >90% agreement in the first round of pilot-testing. After reaching a full team agreement, each team member extracted the study data independently. The data extraction form can be found in the published protocol paper [36]. Subsequently, the studies were divided among the team to be abstracted by a single team member and verified by a second reviewer (FT and AA) to ensure data accuracy. Consistent with a scoping review approach, the methodological quality of the included articles was not appraised [41].

### Stage 5: Data Summary and Synthesis of Findings

The charted data were summarized to gain a descriptive understanding of the data collected. In addition, to answer the 3 subquestions, we used the qualitative content analysis method (Textbox 4).

**Qualitative content analysis method.**

- The technologies described in the identified studies were categorized using the eHealth Enhanced Chronic Care Model (eCCM) [21] to assess the types of functions and technological components supporting chronic disease management for patients with complex care needs in integrated primary care settings. We used a deductive content analysis method [42] to categorize the identified information and communications technologies according to the eCCM domains. The key definitions of the eCCM that we used in the analysis are described in Textbox 2.
- To understand how technologies supported integrated care-related activities, the included studies were thematically mapped onto the Rainbow Model of Integrated Care (RMIC) [13]. A deductive content analysis [42] was conducted to analyze the study data and was linked to the 6 dimensions of integrated care included in Textbox 1. In addition, we extracted the measured outcomes of each study as the RMIC suggested that integrated care should lead to outcomes aligned with the triple aim, which are lower cost, improved health, and improved care [13].
- To explore the implementation factors reported in the identified studies, we used an inductive content analysis method [42].

### Stage 6: Knowledge Dissemination

The study findings have been disseminated in academic conferences [43] and a webinar [44]. A wide variety of stakeholders, such as clinicians, researchers, policy makers, and patients, some of whom provided feedback and reflections on the findings, participated in both academic conferences and the webinar. This manuscript marks another key knowledge dissemination tool for this study.

## Results

### Overview

**Figure 1:**
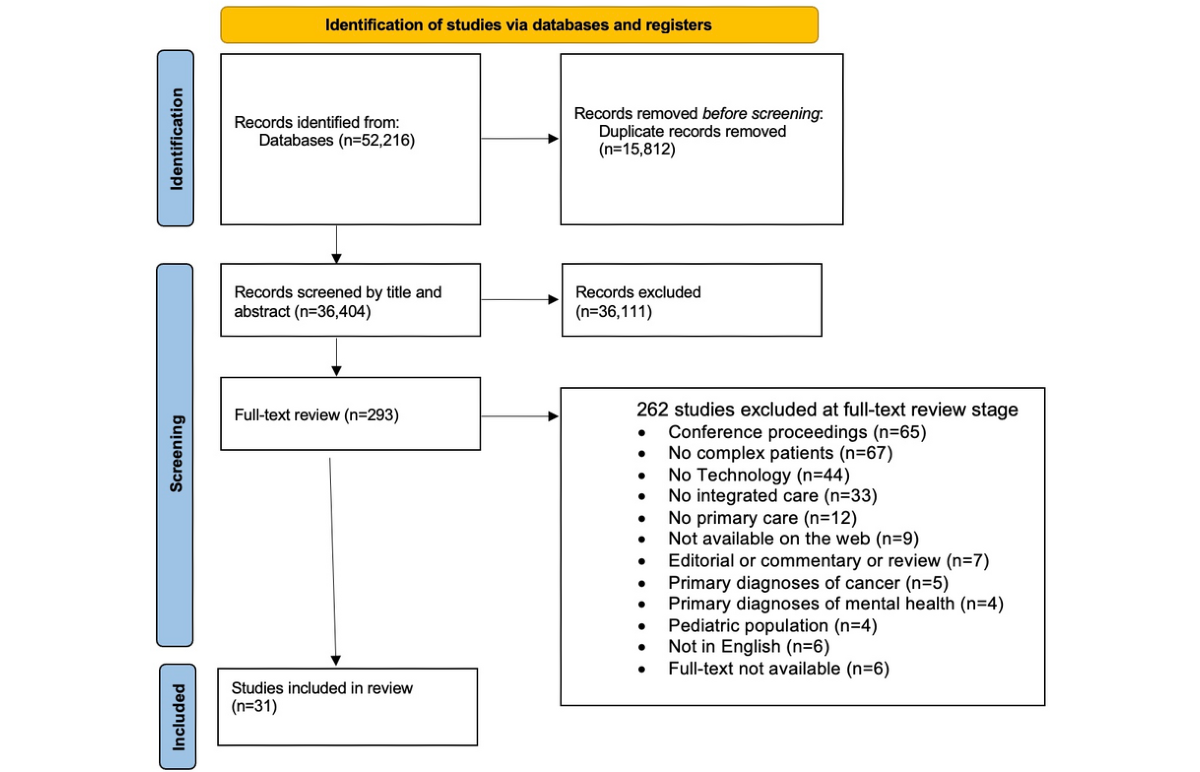
PRISMA (Preferred Reporting Items for Systematic Reviews and Meta-Analyses) flow diagram [44].

A total of 52,286 articles were identified. After removing duplicates, 69.76% (36,474/52,286) of the articles underwent title and abstract screening. A total of 293 articles underwent a full-text review, resulting in 31 (10.6%) that met the eligibility criteria and were included in the final analysis. Figure 1 shows the PRISMA (Preferred Reporting Items for Systematic Reviews and Meta-Analyses) flow diagram for this review.

### Study Characteristics

Table 1 shows the full description of each study. The included articles spanned nearly 16 years (2003-2020), with 68% (21/31) published after 2014 (Table 2). Of the 31 included studies, 15 (48%) were from the United States, with the remaining 52% (16/31) being from Canada, the United Kingdom, Spain, Israel, Taiwan, and India. Of the 31 selected studies, 15 (48%) were quantitative, whereas 5 (16%) used qualitative methods, and 3 (10%) used mixed methods research designs.

**Table 1:** Description of the included studies (N=31).

Study, year	Method	Country	Targeted chronic condition	ICT^a^ component of the intervention	Type of provider involved	Nature of technology^b^	Type of primary care	Type of patient	Outcomes
Alkema et al [46], 2003	Case study	United States	N/A^c^	Telephone	SW^d^ and medical case management staff	2-way	Acute care; long-term; community-based; social services; PCP^e^	Frail older adults enrolled in the Medicare program	N/A
Alkema et al [47], 2007	Quantitative; RCT^f^	United States	Cancer, COPD^g^ and pneumonia, diabetes, fractures, heart disease, hypertension, kidney disease, osteoarthritis, prostate cancer, and stroke	Telephone	Care advocates, PCPs, specialists, and home and community care service providers	2-way	Acute care; long-term; community-based; social services; PCP	Frail older adults enrolled in the Medicare program	Quality of life, mortality, and fewer hospitalizations
Allen et al [48], 2011	Quantitative; RCT	United States	COPD, diabetes, hypertension, chronic HF^h^, osteoporosis, and osteoarthritis	Telephone	Geriatrician, nurse care manager, advanced practice nurse, SW, and geriatrics-certified pharmacist; extended team experts included a psychologist, cardiologist, pulmonologist, endocrinologist, and occupational therapist	2-way	Hospital; social services; primary care	Older adults who were eligible for Medicare and Medicaid	Quality of life, physical condition, self-efficacy, behavior change, caregiver strain, empowerment, quality effectiveness, fewer hospitalizations, other use, access to care, and satisfaction
Allen et al [49], 2012	Quantitative; pilot RCT study	United States	N/A	Electric protocol template, telephone, and fax	Care managers, a physician (PCP), a palliative care physician and specialist, a geriatrician, a SW, a spiritual advisor, and a pharmacist; extended team included physical and occupational therapists, a dietitian, a geriatric advance practice nurse, and a psychologist	2-way	Acute hospital care with community service; PCP	Older adults who were eligible for Medicaid, had a life-limiting illness and 2 deficits in activities of daily life, and were aged >60 years	Quality of life, symptom management, patient-provider relationships, and anxiety and depression
Alyousef et al [50], 2017	Quantitative survey; cross-sectional study	United States	N/A	Case management software, EHRs^i^, and HIE^j^	Care managers and other health care providers	1-way	Outpatient clinics and a transition-of-care center but works with primary care case management software	Patients with chronic conditions	Workload, burnout, and intention to use
Bendixen et al [51], 2007	Case report	United States	Arthritis, diabetes, hypertension, and pulmonary disease	Basic computer and cell phone with internet capability and in-home messaging device	Occupational therapists, an IT specialist, and a program support assistant	2-way	PCP; rehabilitation center	Patients at high risk with complex chronic conditions	Quadruple aim, cost-efficiency, usability, and acceptability
Berry et al [52], 2013	Institutional case study	United States	N/A	EMR^k^	Care coordinators, nurses, SWs, and specialized nurses	1-way	Primary and specialty care	Older adults with multiple conditions	Quality effectiveness and less hospitalizations
Darkins et al [30], 2008	Case report	United States	Diabetes mellitus, chronic HF, hypertension, PTSD^l^, COPD, and depression	Telehealth, which included SMS text messages and biometric devices	Nurses or SWs	2-way	Primary ambulatory care	Older adults with chronic disabling conditions	Patient satisfaction and hospital admissions
Gagnon et al [53], 2019	Mixed methods; cluster randomized trial	Canada	At least 2 of the following: diabetes, hypertension, and dyslipidemia	Personalized eHealth platform named CONCERTO+	PCP	Unclear	Primary care	Patients with chronic conditions	Patient activation measure and technology acceptance
Gutteridge et al [54], 2014	Quantitative; descriptive study	United States	N/A	Clinical event notification	Geriatric ED^m^ physicians, transitional care NPs^n^, PCPs, SWs, geriatric ED pharmacists, physical therapists, and CARE^o^ volunteers	1-way	ED; PCP integration	Older adults who were triaged to a bed in the geriatric ED	Use of the technology
Hans et al [55], 2018	Qualitative; descriptive study	Canada	N/A	Goal management app	PCPs, nurses, and allied health professionals	1-way	Primary care	Individuals living with chronic illness or disability or living with multimorbidity	N/A
Hernandez et al [56], 2015	Comparative case study	Multiple countries: Spain, Norway, and Greece	COPD and respiratory symptoms	Remote diagnosis of spirometry testing (not described fully)	N/A	Unclear	ED	Patients who visited primary care for respiratory symptoms	Accessibility to testing
Jindal et al [57], 2018	Developmental pilot study	India	Cardiovascular disease and diabetes	mHealth^p^ app	Nurse, PCP, and pharmacist	2-way	Primary health care	Patients with cardiovascular disease and diabetes	N/A
Marek et al [58], 2013	RCT	United States	N/A	Medication dispensing system with reminders	Advanced practice nurses, RNs^q^, multiple physicians, pharmacies, social service agencies, and others	1-way	N/A	Patients with complex medication regimens for chronic illnesses	N/A
Martín-Lesende et al [59], 2013	Quantitative; RCT	Spain	HF or chronic lung disease	Telemonitoring of patients’ clinical parameters and PDA platform	Health professionals, nurses, and GPs^r^	1-way	Primary care	HF or chronic lung disease	The number and length of hospital admissions
Martín-Lesende et al [60], 2011	Quantitative; RCT	Spain	HF or chronic lung disease	Telemonitoring of self-measured clinical parameters that were measured using Bluetooth wireless sensors	Primary care nurses, GPs, and hospital providers	1-way	Primary care	HF or chronic lung disease	The number and length of hospital admissions
Martín-Lesende et al [61], 2017	Quantitative; uncontrolled before-and-after study	Spain	HF and CLD^s^ (mainly COPD and a few with asthma)	Telecare	GP and nurse	1-way	Primary care	N/A	Rate of hospital use
May et al [62], 2011	Qualitative; comparative study	England	Asthma, coronary heart disease, COPD, and diabetes	Telecare system	PCPs and SWs	N/A	Primary care	Patients with multiple chronic conditions	N/A
Metting et al [63], 2018	Qualitative; exploratory study	The Netherlands	Asthma and COPD	Patient web portal	N/A	1-way	Primary care	Patients with asthma and COPD from low socioeconomic areas	N/A
Noel et al [64], 2018	Quantitative; superior RCT with parallel group	United States	N/A	Smartphone device and Bluetooth-enabled clinical parameter measurement	Senior resident physician (“teledoc”) and family medicine PCPs	1-way	Primary and social care	Multi-comorbid conditions	30-day hospital readmissions
Porath et al [65], 2017	Quasi-experimental; observational study	Israel	Diabetes, hypertension, transient ischemic attack, HF, COPD, and dementia	Web-based clinical protocols	MTC^t^ personal nurse, PCP, other physician consultants (cardiologists, pulmonary specialists, endocrinologists, and psychogeriatricians), clinical pharmacists, SWs, nutritionists, and an administrative team	1-way	Inpatient and ambulatory care	Frail older adults with chronic conditions	Hospital use and hospitalization cost
Portz et al [66], 2018	Qualitative descriptive	United States	Multiple chronic conditions	Patient portal	PCP	1-way	Primary health care	Patients with multiple chronic conditions	N/A
Price-Haywood et al [67], 2017	Quantitative; retrospective observational study	United States	Diabetes and hypertension	Shared web-based portal	PCP	Unclear	Primary care	Older adults with diabetes or hypertension	N/A
Ryan et al [68], 2003	Quantitative; evaluative pilot study	United States	Diabetes, hypertension, schizophrenia, and depression	An in-home messaging device, the Health Buddy; a disease management app; Instamatic cameras for diabetic wound care management; and personal computers with internet connectivity for supervised chat rooms	Care coordinators and other unspecified providers	2-way	Community-based primary care clinics; hospitals; outpatient clinics	Patients with multimorbidity	Patient and provider satisfaction, compliance, and quality of life
Sorocco et al [69], 2013	Quantitative; pilot study	United States	COPD, spinal cord injury, diabetes, hypertension, syncope, depression, dementia, anxiety, and PTSD	Video telehealth (mental health and occupational therapy) and remote monitoring of vitals using a digital scale	Physician assistant, nurse or NP, pharmacist, nutritionist, occupational therapist, SW, and mental health practitioner	2-way	Primary care	Veterans with complex medical conditions	Satisfaction and mental health conditions
Steele Gray et al [70], 2019	Multimethods; exploratory trial	Canada	N/A	ePRO^u^ tool, a goal-supporting mHealth app	Physician, NP, nurse, SW, and dietitian	1-way	Primary care	Patients aged ≥60 years living with 2 or more chronic conditions and provider referred	Quality of life, cost-effectiveness, experience of care, and attainment of goals
Steele Gray et al [71], 2016	Qualitative; descriptive interpretive study	Canada	N/A	ePRO tool, a goal-supporting mHealth app	PCPs, NPs, RNs, SWs, and dietitians	1-way	Primary care	Patients aged ≥60 years living with 2 or more chronic conditions and provider referred	N/A
Steele Gray et al [70], 2016	Mixed methods; RCT, cost-effectiveness analysis, and research protocol	Canada	N/A	ePRO tool, a goal-supporting mHealth app	PCPs, NPs, RNs, SWs, and dietitians	1-way	Primary care	Patients aged ≥60 years living with 2 or more chronic conditions and provider referred	Quality of life, cost-effectiveness, experience of care, and attainment of goals
Uei et al [72], 2017	Quantitative; cross-sectional study	Taiwan	Diabetes or hypertension	Telephone and patient health record	N/A	2-way	Primary care	Individuals with multiple chronic conditions	Telecare use intention
Valdivieso et al [73], 2018	Quantitative; RCT	Spain	N/A	Tablet with Bluetooth connection that receives information from digital health scales, EHR, and patient portal	Primary care team and hospital case manager nurses	2-way	Primary care	Older adult patients at high risk	Quality of life, mortality, and health care use
Varey et al [74], 2020	Qualitative	England	General chronic conditions	Tablet	NP, clinical care coordinator, primary care assistant, and nonclinical team leader	1-way	Primary care	Older adults with chronic conditions	N/A

^a^ICT: information and communications technology.

^b^1-way versus 2-way: ICTs that support 1-way communication typically transmit biomarkers, vital signs, health information, and educational messages [75,76]. In contrast, ICTs that support 2-way communication facilitate conversations between patients and clinicians via telephone and SMS text messaging [75,76].

^c^N/A: not applicable.

^d^SW: social worker.

^e^PCP: primary care physician.

^f^RCT: randomized controlled trial.

^g^COPD: chronic obstructive pulmonary disease.

^h^HF: heart failure.

^i^EHR: electronic health record.

^j^HIE: health information exchange.

^k^EMR: electronic medical record.

^l^PTSD: posttraumatic stress disorder.

^m^ED: emergency department.

^n^NP: nurse practitioner.

^o^CARE: Care and Respect for Elders.

^p^mHealth: mobile health.

^q^RN: registered nurse.

^r^GP: general practitioner.

^s^CLD: chronic liver disease.

^t^MTC: Maccabi Telecare Center.

^u^ePRO: electronic patient-reported outcomes.

**Table 2:** Total number of publications by year (N=31).

Publication year	Articles, n (%)
2000	0 (0)
2001	0 (0)
2002	0 (0)
2003	2 (6)
2004	0 (0)
2005	0 (0)
2006	0 (0)
2007	2 (6)
2008	1 (3)
2009	0 (0)
2010	0 (0)
2011	3 (10)
2012	1 (3)
2013	4 (13)
2014	1 (3)
2015	1 (3)
2016	2 (6)
2017	5 (16)
2018	6 (19)
2019	2 (6)
2020	2 (6)
2021	1 (3)

### Intervention Settings

Most of the interventions (15/31, 48%) [14,53, 55,57,59-63,66,69-71,77,78] only took place in a primary health care setting. However, 39% (12/31) of the studies linked primary care to acute care [30,46-49,52,54,56,65,68], social services [46-48,64], or rehabilitation centers [51].

### Health Care Providers

Of the 31 identified studies, 17 (55%) described the involvement of primary care physicians [47,49,53-55,57-61,64-66,70, 71,77,78]. A total of 52% (16/31) of the studies described the involvement of registered and specialized nurses, such as geriatric nurses [30,48,49,52,54,55,57,58,60,61,65,70,71,73, 74,78]. In addition, 23% (7/31) of the studies described the involvement of nonclinical members such as care coordinators and care managers [46,47,49-52,68].

### Types of ICTs

#### Overview

The most prominent type of technology discussed across the identified studies (21/31, 68%) were ICTs that support patients’ health status monitoring by providers, which are also known as telemonitoring devices [30,53-65,68-71,73,74,78]. Telemonitoring devices include mobile health (mHealth) apps where patients can either actively input their health status or automated biometric (eg, blood pressure) devices connected to a smartphone, tablet, or web-based application can receive that information [30,51,55,64,68,70,73,74,78]. This sort of telemonitoring device served dual purposes: (1) enabling physicians to continuously monitor patients and (2) facilitating patient self-management, meaning that patients can track their own symptoms and disease progression. Electronic health records (EHRs) were described in 23% (7/31) of the studies to share patient data across multiple professionals or organizations (eg, acute care hospitals and primary care) [50,52,63,66,72,73,77]. Telephone-supported interventions were discussed in 16% (5/31) of the studies to facilitate patient follow-up remotely [46-50].

#### ICTs Aligned With the eCCM

Table 3 shows the ICTs categorized into the eCCM and RMIC models. Of the 31 studies, 24 (77%) described ICTs that supported the *clinical information management* aspect of the eCCM to provide relevant patient data to one or more health care providers [30,48-51,53-55,57-61,63-65,68,69,72-74,78]. The types of ICTs for clinical information management were wide-ranging: smartphone apps, telemonitoring devices, patient health records (PHRs), and telephones. To support clinical information management, these ICTs relied on patient self-reporting of symptoms and health status and on providers to monitor patient data remotely. The clinical information management functions of ICTs were important as shared patient information supported other key functions of the Chronic Care Model such as self-management and delivery system design.

Remote patient monitoring data enabled the *delivery system design* aspect of the eCCM as health professionals followed up with patients to provide additional support or consultation based on patient data [30,46-52,54-57,62,64,68,70-73,78]. In total, 65% (20/31) of the studies described ICTs that enhanced *delivery system design* in the integrated primary care model by sharing data across team members, communicating about patients’ health status, and facilitating regular team meetings [30,46-52,54-57,62,64,68,70-73,78]. Telephones, EHRs, mHealth apps, and case management software were used to enhance the delivery system [30,46-52,54-57,62,64,68, 70-73,78].

A total of 61% (19/31) of the studies described ICTs that supported the patient *self-management* aspect of the eCCM by engaging patients in symptom monitoring and tracking. mHealth apps or web-based platforms, medication reminders through medication adherence devices, and providing patients with their health data and appointments through PHRs were used to support patient self-management [30,48,49,51,53, 55,57-62,66,70,72,74,77,78]. In all 61% (19/31) of the studies, the primary care team had access to patients’ self-management–related data.

A total of 16% (5/31) of the studies described ICTs such as case management software, EHRs, and mHealth that facilitate evidence-based and data-driven *clinical decision-making* aspects of the eCCM [30,48,49,57,65]. The ICTs enabled the clinical decision-making aspect of the eCCM by being a platform for clinical algorithms that alert or remind health professionals to follow up with patients [30,48,49,57]. The clinical algorithms were developed based on evidence-based clinical guidelines for providers to make decisions about how to support patients with chronic conditions remotely [30,48,49,57]. None of the studies explicitly described ICTs that support the eCommunity resource aspect of the eCCM.

The eCCM emphasizes the importance of productive interactions between patients and providers to ensure mutual partnership and communication. According to Pols [75], health-related ICTs can be divided into 2 broad categories: ICTs supporting 1-way versus 2-way communication [75]. ICTs that support 1-way communication typically transmit biomarkers, vital signs, health information, and educational messages [75,76]. In contrast, ICTs that support 2-way communication facilitate conventional conversations between patients and clinicians via telephone and SMS text messaging [75,76]. In this review, 35% (11/31) of the studies described an ICT that enabled 2-way communication between patients and providers [46-49,51,57,65,68,69,72,73]. This 2-way communication was supported by ICTs such as telephones, internet-enabled computers, smartphones, and fax. In contrast, ICTs such as telemonitoring devices, electronic medical records (EMRs), patient portals, and mHealth apps supported 1-way communication features in 48% (15/31) of the studies, allowing providers to receive patient information remotely through ICTs [50,52,54,55,59-61,64,70,71,78] and patients to receive automated algorithm-generated reminders about medications or lifestyle advice related to chronic conditions [58,63,64,74].

**Table 3:** Categorization into the Rainbow Model of Integrated Care and eHealth Enhanced Chronic Care Model of the included studies.

Study, year	Type of technology	Delivery system design	Self-management support enhancements	Clinical decision support enhancements	Clinical information system enhancements	eHealth education	Clinical	Professional	Organizational
Alkema et al [46], 2003	Telephone	✓					✓		✓
Alkema et al [47], 2007	Telephone	✓					✓		✓
Allen et al [48], 2011	Telephone	✓	✓	✓	✓		✓	✓	✓
Allen et al [49], 2012	Electric protocol template, telephone, and fax	✓	✓	✓	✓		✓	✓	✓
Alyousef et al [50], 2017	Case management software, EHR^a^, and HIE^b^	✓			✓		✓	✓	
Bendixen et al [51], 2007	Basic computer and cell phone with internet capability and in-home messaging device	✓	✓		✓		✓		
Berry [52], 2003	EMR^c^	✓			✓			✓	✓
Darkins et al [30], 2008	Telehealth, including SMS text messages and biometric devices	✓	✓	✓	✓		✓	✓	
Gagnon et al [53], 2019	Personalized eHealth named CONCERTO+		✓		✓		✓		
Gutteridge et al [54], 2014	Clinical event notification	✓			✓			✓	✓
Hans et al [55], 2018	Goal management app	✓	✓		✓		✓	✓	
Hernandez et al [56], 2015	Remote diagnosis of spirometry testing (not described fully)	✓					✓		
Jindal et al [57], 2018	mHealth^d^ app	✓	✓	✓	✓		✓	✓	
Marek et al [58], 2013	Medication dispensing system with reminders		✓		✓	✓	✓		
Martín-Lesende et al [59], 2013	Telemonitoring of patients’ clinical parameters and PDA platform		✓		✓		✓	✓	
Martín-Lesende et al [60], 2011	Telemonitoring of self-measured clinical parameters		✓		✓		✓	✓	
Martín-Lesende et al [61], 2017	Telecare		✓		✓		✓	✓	
May et al [62], 2011	Telecare system	✓	✓						✓
Metting et al [63], 2018	Patient web portal				✓		✓		
Noel et al [64], 2018	Smartphone device and Bluetooth-enabled clinical parameter measurement	✓			✓		✓		
Porath et al [65], 2017	Remote consultation using telecare			✓	✓	✓	✓	✓	
Portz et al [66], 2020	Patient portal		✓	✓				✓	
Price-Haywood et al [67], 2017	Shared web-based portal		✓		✓				
Ryan et al [68], 2003	An in-home messaging device, the Health Buddy; a disease management app; Instamatic cameras for diabetic wound care management; and personal computers with internet connectivity for supervised chat rooms	✓			✓		✓		✓
Sorocco et al [69], 2013	Video telehealth and remote monitoring of vitals using a digital scale	✓			✓		✓	✓	
Steele Gray et al [70], 2019	ePRO^e^ tool, a goal-supporting mHealth app	✓	✓		✓		✓	✓	
Steele Gray et al [71], 2016	ePRO tool, a goal-supporting mHealth app	✓	✓		✓		✓	✓	
Steele Gray et al [70], 2016	ePRO tool, a goal-supporting mHealth app	✓	✓				✓	✓	
Uei et al [72], 2017	Telephone and patient health record	✓	✓		✓			✓	
Valdivieso et al [73], 2018	Tablet with Bluetooth connection that receives information from digital health scales, EHR, and patient portal	✓			✓	✓	✓	✓	
Varey et al [74], 2019	Tablet		✓		✓		✓		

^a^EHR: electronic health record.

^b^HIE: health information exchange.

^c^EMR: electronic medical record.

^d^mHealth: mobile health.

^e^ePRO: electronic patient-reported outcomes.

#### ICTs Aligned With the RMIC

A total of 77% (24/31) of the studies described ICTs that enabled *clinical or service integration* by coordinating clinical services across multiple professionals, organizations, and sectors (ie, long-term care or acute care services with primary care services) [30,46-51,53,55-61,63-65,68,70,71,73,74,78]. mHealth apps, web-based platforms, disease-specific self-measurement devices (eg, glucose monitoring devices), case management software, EMRs, and PHRs were used to ensure care continuity across multiple disciplines. These wide-ranging ICTs supported collaborative goal setting between patients and providers through the use of mHealth apps [55,57,70,78]; coordination of patient information and clinical guidelines through EHRs, software, or case management platforms [50,58-61,65]; connecting patients with multiple services by using telephone consultation [46,47]; and supporting patient self-management by using mHealth and telephones [57,58].

In total, 61% (19/31) of the studies described ICT-enabled *professional integration* among multidisciplinary teams within the same organization or professionals across multiple organizations [30,48-50,52,54,55,57,59-61,65,66,69-73,78]. ICTs such as case management platforms, telemonitoring devices, and EHRs or patient portals were used for clinical information sharing across team members [30,59-61,65]. This shared information was considered important for service coordination and delivery to patients. In addition, mHealth devices such as smartphones and tablets were used to transmit patient data to multiple providers simultaneously [55,70,78]. In 19% (6/31) of the studies, an assigned team member (nurse, social worker, or general practitioner) followed up with patients for a routine checkup or to provide health education [59-61,64,65,72].

A total of 26% (8/31) of the studies described *organizational integration*, achieved through the data-sharing feature of ICTs [46-49,52,54,62,68]. In total, 16% (5/31) of the studies pursued organizational integration through a shared EHR or case management platform, and patient information was shared across multiple organizations [49,52,54,62,68]. A total of 10% (3/31) of the studies supported organizational integration by creating connections between primary care teams and community services [46-48]. We did not find any studies that pursued system-level integration.

The “formative aspect” of integration is described as the extent to which key functions in a health care model are coordinated across time, services, and settings [13]. The reviewed articles in this study describe multiple key functions achieved through ICTs in integrated primary care settings. The articles emphasized how the coordination of care and information sharing between professionals and between organizations are achieved through ICTs. Similarly, the reviewed articles identified the role that ICTs play in the self-management of chronic conditions supporting patients with complex care needs. In contrast, as the normative aspect of integrated care is achieved through functional, organizational, and service integration, typically, the normative aspect of integration is not considered as a stand-alone objective of an intervention [18,79]. Rather, normative integration is considered as a process or mechanism of integration [18]. Hence, in this study, we did not identify any examples of how technologies influenced the values, attitudes, and beliefs of actors, organizations, and systems, which are some components of normative integration.

#### The Types of ICTs and Their Role in Supporting Functions of eCCM and Enabling RMIC

Table 4 shows how different types of ICTs support different functions of the eCCM and enable different levels of the RMIC. Telemonitoring devices supported self-management and clinical information system components of the eCCM in 48% (15/31) of the studies [30,51,53,56,59-62,64,65,68,69,72-74]. The telemonitoring devices often included the daily or weekly reception of patient data shared across teams and organizations and routine follow-up by providers [30,51,53,56,59-62,68,69,72-74]. This shared patient information enabled clinical, professional, and organizational integration. In addition, by engaging patients in tracking and monitoring their own conditions, these telemonitoring devices enabled self-management support among patients. In 10% (3/31) of the studies, EHRs or EMRs enabled sharing of patient data across multidisciplinary teams [49,50] and organizations [52], which enabled professional- and organizational-level integration, respectively. In addition, in 10% (3/31) of the studies, patient health portals were described as a tool for patients to access their own health records or appointments and contact health professionals via email [63,66,67]. These patient portals enabled clinic- or service-level integration through clinical information management [63,66,67]. mHealth apps supported patient self-management to track or monitor patients’ health, social goals, and symptoms as well as share patient information across multidisciplinary teams to tailor clinical services to patients based on their clinical information [55,57,70,71,78]. Finally, 10% (3/31) of the studies described the use of telephones to provide patient education and follow-up and link patients with community resources [46,48,49].

**Table 4:** Categorization into the Rainbow Model of Integrated Care and eHealth Enhanced Chronic Care Model of the included studies.

Study, year		Type of technology and its function	Delivery system design	Self-management support enhancements	Clinical decision support enhancements	Clinical information system enhancements	eHealth education	Clinical	Professional	Organizational
**Telephone**										
	Alkema et al [46], 2003	To receive social care management from social workers	✓					✓		✓
	Alkema et al [47], 2007	To link patients with source and for monitoring and follow-up by social workers	✓					✓		✓
	Allen et al [48], 2011	Patient education and monitoring by nurses	✓	✓	✓	✓		✓	✓	✓
**EHR^a^, EMR^b^, and PHR^c^**										
	Allen et al [49], 2012	Electric protocol template, telephone, and fax for patient monitoring and evidence-based guidelines	✓	✓	✓	✓		✓	✓	✓
	Alyousef et al [50], 2017	Case management software, EHR, and HIE^d^ to facilitate patient information across professionals and organizations	✓			✓		✓	✓	
	Berry et al [52], 2003	EMR for coordinating multiple services provided to patients	✓			✓			✓	✓
	Metting et al [63], 2018	Patient web portal: a place for patients to access their information				✓		✓		
	Portz et al [66], 2020	Patient portal: a place for patients to access their information		✓				✓		
	Price-Haywood et al [67], 2017	Shared web-based portal: a place for patients to access their information		✓		✓				
**Telemonitoring devices**										
	Bendixen et al [51], 2007	Basic computer and cell phone with internet capability and in-home messaging device for daily patient monitoring and communication between patient and provider	✓	✓		✓		✓		
	Darkins et al [30], 2008	Telehealth, including SMS text messages and biometric devices, to monitor patients’ vital signs remotely and coordinate care accordingly	✓	✓	✓	✓		✓	✓	
	Gagnon et al [53], 2019	Personalized eHealth named CONCERTO+ for sharing patient self-reported data with multiple providers and organizations		✓		✓		✓		
	Hernandez et al [56], 2015	Remote diagnosis of spirometry testing (not described fully)	✓					✓		
	Martín-Lesende et al [59], 2013	Telemonitoring of patients’ reported clinical parameters, PDA platform, and routine follow-up via phone		✓		✓		✓	✓	
	Martín-Lesende et al [60], 2011	Telemonitoring of self-measured clinical parameters and routine follow-up via phone		✓		✓		✓	✓	
	Martín-Lesende et al [61], 2017	Telemonitoring of self-measured clinical parameters and routine follow-up via phone		✓		✓		✓	✓	
	May et al [62], 2011	Telecare system: general patient perception of any telehealth-related services	✓	✓						✓
	Noel et al [64], 2018	Telehealth: smartphone device, Bluetooth-enabled clinical parameter measurement by patients daily, and weekly follow-up by providers	✓			✓		✓		
	Porath et al [65], 2017	Telemonitoring system for patient monitoring, patient education, and coordination of multiple services			✓	✓	✓	✓	✓	
	Ryan et al [68], 2003	Several technologies: (1) an in-home messaging device, the Health Buddy; (2) a disease management app; (3) Instamatic cameras for diabetic wound care management; and (4) personal computers with internet connectivity for remote consultation and supervised chat rooms	✓			✓		✓		✓
	Sorocco et al [69], 2013	Telehealth: remote monitoring of daily vitals using a digital scale and provision of services remotely via video	✓			✓		✓	✓	
	Uei et al [72], 2017	Telehealth (telephone and PHR) for monitoring patient health status and providing health education	✓	✓		✓			✓	
	Valdivieso et al [73], 2018	Telehealth: tablet with Bluetooth connection that receives information from digital health scales that is connected to EHR and patient portal	✓			✓	✓	✓	✓	
	Varey et al [74], 2019	Motiva clinical system: a tablet or television where patients can report their vital signs and providers can give educational content, messages, and reminders		✓		✓	✓	✓		
**Clinical event notifications (for providers)**										
	Gutteridge et al [54], 2014	Clinical event notification to inform multiple health professionals about patients’ discharge and schedules to coordinate care across organizations and providers	✓			✓			✓	✓
**Medication reminder for patients**										
	Marek et al [58], 2013	Medication dispensing system with reminders		✓		✓	✓	✓		
**mHealth^e^ apps**										
	Hans et al [55], 2018	Goal management app	✓	✓		✓		✓	✓	
	Jindal et al [57], 2018	mHealth app	✓	✓	✓	✓		✓	✓	
	Steele Gray et al [70], 2019	ePRO^f^ tool, a goal-supporting mHealth app	✓	✓		✓		✓	✓	
	Steele Gray et al [71], 2016	ePRO tool, a goal-supporting mHealth app	✓	✓		✓		✓	✓	
	Steele Gray et al [70], 2016	ePRO tool, a goal-supporting mHealth app	✓	✓				✓	✓	

^a^EHR: electronic health record.

^b^EMR: electronic medical record.

^c^PHR: patient health record.

^d^HIE: health information exchange.

^e^mHealth: mobile health.

^f^ePRO: electronic patient-reported outcomes.

### Outcomes and Process Measures Reported in the Studies

Aligning with the RMIC goal of improving the patient experience by pursuing the triple aim of health care, the studies reported outcomes related to the triple aim, such as quality of life (improved health), cost-effectiveness (lower cost), and acceptability of the care delivery system (improved care). Outcome measures included patients’ health outcomes (eg, mental and physical conditions), patient and provider perceived outcomes (eg, satisfaction), and cost-effectiveness. Patient experience measures included satisfaction, perceived empowerment, caregiver strain, loneliness, self-efficacy, acceptability, and usability of the technology [19,30,48-51,68-70,72,78]. Cost-effectiveness included costs associated with hospital use and cost-efficiency [30,47,48,51,52,59-61,64,65,73,78]. We did not observe any pattern in the type of outcome or technology.

In total, 68% (21/31) of the studies evaluated outcomes. A total of 45% (14/31) measured selected clinical outcomes such as quality of life (6/31, 19%) [47-49,70,73,78,80], mental or physical conditions [49,69], and hospital use (10/31, 32%) [30,47,48,52,60,61,64,65,73]. Among the nonclinical outcomes, provider workload [50], patient-provider relationship [49], patient satisfaction [30,48,69,80], cost-effectiveness [51,65,78], and attitude toward technologies [50,51,53] were studied. In contrast, patients’ self-efficacy (3/31, 10%) [48,53,78] and acceptability of the technologies (4/31, 13%) [50,51,53,72] were explored mostly in mixed methods and qualitative studies. The cost-effectiveness (4/31, 13%) [51,65,78] of the implemented technologies was evaluated quantitatively and through mixed methods research.

### Implementation Factors Reported in the Identified Studies

The implementation of technology has been identified in the digital health literature as a key issue that requires further exploration [81]. Thus, we extracted the factors associated with the successful implementation of technology in the models of care described in the studies. Inductive analysis of the studies revealed implementation factors of technologies that were categorized into the patient, provider, technology, and organizational level. Table 5 shows the implementation factors discussed in each study.

Patient characteristics that were described as important implementation factors in the selected studies included age, gender, type of chronic conditions [46,64], patients’ interest in buying into the technology [14], digital literacy [59,63], and perceived ease of use and usefulness [66]. At the provider level, their willingness to adopt digital technology in their practice [55,59,71,80], collaboration and communication among providers [56,57], and providers’ level of ICT training [64] contributed to the implementation of ICT-enabled integrated primary care. Barriers to implementation arose when technology disrupted provider workflow [55,69,71] and increased their workload [71].

Some implementation factors spanned more than one category. Notably, the patient-provider relationship emerged as an important factor [52,55]. For example, Berry et al [52] signaled that technology-enabled care works better when there is a long-established trusting relationship between the patient and the provider.

In addition, technology- and organizational-related implementation factors emerged. Technology-related factors such as system errors [54,55,63,71], lack of interoperability with existing technologies (such as EMRs) [55,69,71], and technologies’ lack of responsiveness to the patient and providers’ needs and tasks were found to deter implementation in 16% (5/31) of the studies [50,55,69,70,78]. At the organizational level, funding support [65] and organizations’ willingness to adopt a new technology [14] were reported as important organizational-level implementation factors. Moreover, having a coordinator to support IT-related issues [63], having a team structure that involves nurses [59], and having additional care coordination [52] were also reported as important implementation factors for ICT-based integrated primary care.

**Table 5:** Presence and absence of implementation factors.

	Patient	Provider	Technology	Organizational
Alkema et al [46], 2003				✓
Alkema et al [47], 2007	✓			✓
Allen et al [48], 2011	✓	✓		✓
Allen et al [49], 2012	✓	✓		
Alyousef et al [50], 2017			✓	
Bendixen et al [51], 2007	✓	✓	✓	✓
Berry et al [52], 2013	✓	✓	✓	
Darkins et al [30], 2008			✓	
Gagnon et al [53], 2019		✓	✓	
Gutteridge et al [54], 2014	✓			
Hans et al [55], 2018	✓	✓		
Hernandez et al [56], 2015			✓	✓
Jindal et al [57], 2018				
Marek et al [58], 2013	✓	✓	✓	✓
Martín-Lesende et al [59], 2013				
Martín-Lesende et al [60], 2011		✓	✓	
Martín-Lesende et al [61], 2017	✓	✓	✓	✓
May et al [62], 2011		✓		✓
Metting et al [63], 2018	✓	✓	✓	✓
Noel et al [64], 2018		✓		✓
Porath et al [65], 2017		✓	✓	✓
Portz et al [66], 2018	✓			
Price-Haywood et al [67], 2017	✓	✓		
Ryan et al [68], 2003			✓	✓
Sorocco et al [69], 2013		✓	✓	
Steele Gray et al [70], 2019	✓	✓	✓	✓
Steele Gray et al [71], 2016	✓	✓	✓	✓
Steele Gray et al [70], 2016	✓	✓	✓	✓
Uei et al [72], 2017	✓			
Valdivieso et al [73], 2018		✓		
Varey et al [74], 2020	✓		✓	✓

## Discussion

### Principal Findings

The primary objective of this scoping review was to provide a comprehensive overview of the types of ICTs that are being used to support patients with complex care needs in the integrated primary care model. The findings show that, in the current ICT-based integrated primary care models, wide-ranging technologies such as telemonitoring devices, mHealth apps, EHRs, and telephones are being used to support patients with complex care needs. These technologies enable information sharing among multiple professionals within and across organizations and between patients and providers. These technologies support care integration at the service or clinical, professional, and organizational levels. However, system-level integration was absent in this study. The findings also show that patient-, provider-, technology-, and organizational-level factors contribute to the implementation of ICTs in the integrated primary care model.

Among the identified studies, we observed a number of ICTs that enabled clinical information management across teams in the integrated primary care models. These ICTs promoted teamwork across professionals by providing access to critical patient information across the teams. This shared patient information system is considered a critical tenet of an integrated primary care model [13,18]. Moreover, ICTs that supported patient self-management were also frequently observed. This finding is unsurprising as a previous study identified that, given the nature of chronic conditions, self-measurement of the body is frequently used to track symptoms [82]. This self-management approach to chronic conditions can empower patients by engaging them with their care plan, which in turn can improve patients’ health outcomes [83]. In contrast, too many self-management responsibilities can overwhelm patients, especially if the patient has a low capacity (ie, financial resources) to shoulder the responsibilities [10]. For example, a number of ICT-based interventions in this review required daily reporting of patients’ vital signs and symptoms [51,59-61,64,69]. For patients with complex care needs, this daily reporting of vital signs may be overwhelming because of the number of conditions and complexity related to each condition. Therefore, it is critical to examine how to effectively use and design ICTs to minimize the self-management–related workload for patients.

The findings of this study show that a number of ICTs transmit patient data to providers for remote disease monitoring. However, most of these ICTs lack a 2-way communication loop. As a result, providers may receive an overwhelming amount of patient data, but engaging in a productive interaction may be difficult for both the patient and provider without a 2-way communication loop. This is problematic as the quality of the eCCM relies strongly on productive interaction and communication between providers and patients [21]. Therefore, an increased effort to promote meaningful and productive interaction between patients and providers can improve the effectiveness of ICT-based interventions designed for patients with complex care needs [21].

### Identified Research Gap and Recommendation for Future Research

This scoping review revealed several gaps in the current literature. System-level integration and the normative aspect of integration were absent in the sample of studies in this review. Undertaking an ICT-enabled system integration can be challenging because of interoperability issues, provider unwillingness, lack of coalition among stakeholders, lack of data privacy, and security issues [84,85]. For future researchers, it would be important to explore the broader system-level changes that are required for the successful adoption and implementation of ICTs in the integrated primary care model.

In addition, normative aspects of integration, such as culture, shared vision, and regulatory factors, need to be studied further for the long-term sustainability of ICT-based integrated primary care. Evaluation frameworks used in implementation science [86], such as the Consolidated Framework for Implementation Research [87] and the nonadoption, abandonment, scale-up, spread, and sustainability framework by Greenhalgh et al [76], can be useful to explore the normative factors that can contribute to the implementation of ICT-based interventions in the integrated primary care setting.

Furthermore, organizational integration was also minimally described in the studies. Organizational-level integration may be difficult because of a lack of shared vision in terms of policy, regulations, and culture across organizations [18]. This lack of organizational integration may negatively affect patients with complex care needs as they may have to report similar information to multiple providers. For example, although ICTs can be helpful in creating an eCommunity that enables access to patient data for community staff and social service organizations, this aspect of the eCCM is currently underused. However, creating an eCommunity where patients’ clinical data are readily available to formal or informal caregivers can reduce the patients’ need to repeat information to multiple providers, thereby reducing the burden on patients and caregivers [28]. We also identified that the definitions of the components of the eCCM are broad, which made the categorization of the ICTs difficult and interpretive. This criticism of the eCCM has been identified in previous work [88].

In terms of measured outcomes, 45% (14/31) of the studies evaluated the impact of ICTs at the patient level through a clinical lens, such as quality of life. Going beyond clinical outcomes, such as health status or symptoms, and considering the impact of ICTs on patients’ relational factors, such as patient-provider relationships [89] or potential disruption of technology in patients’ lives [5], may be important factors to consider for future researchers.

### Strengths

To categorize the study data, we used 2 established conceptual frameworks, the eCCM and RMIC, which helped identify literature gaps. For example, by categorizing the role of ICTs in the RMIC, we identified that there is little information available about ICT-enabled system-level and normative integration. In addition, the use of these established frameworks will be helpful for future researchers from multiple contexts and jurisdictions to interpret and apply the findings to their own contexts. The use of the scoping review method also allowed us to provide a comprehensive overview of the literature on the topic regardless of methodology, quality of the publications, and measured outcomes [41]. This will be helpful for researchers to gain an understanding of the current landscape of ICTs supporting patients with complex care needs in integrated primary care settings.

### Limitations

This scoping review has a few notable limitations. Owing to time and resource constraints, we were unable to conduct a review of gray literature, potentially missing relevant publications. However, the peer-reviewed sample was extracted from a very large pool of potential articles, which adds to our confidence that the peer-reviewed literature was thoroughly covered. Second, we have observed that the term “complex patient” is defined in various ways in the current literature. We may have missed unexpected characterizations of a patient with complex care needs that are not captured in the current literature. In addition, we did not involve patients or the public during the conceptualization of the research question. However, we tackled this issue by disseminating our research findings among different audiences (conferences and webinars) that were accessible to the public.

### Conclusions

This scoping review identified that multiple types of ICTs play important functional roles in supporting patients with complex care needs in integrated primary care settings. ICTs play a critical role in coordinating multiple clinical services across multidisciplinary teams and supporting the self-management of patients with complex care needs remotely. However, to use the full potential of ICTs, further studies are required to understand how to achieve organizational- and system-level integration.

## Appendix

Multimedia Appendix 1 Key search domains and associated search terms.

Multimedia Appendix 2 Search strategy for PsycINFO.

## Abbreviations

eCCM: eHealth Enhanced Chronic Care Model
EHR: electronic health record
EMR: electronic medical record
ICT: information and communications technology
mHealth: mobile health
PHR: patient health record
PRISMA: Preferred Reporting Items for Systematic Reviews and Meta-Analyses
RMIC: Rainbow Model of Integrated Care
