# Supplementary material for: Information and Communications Technologies Enabling Integrated Primary Care for Patients With Complex Care Needs: Scoping Review
Source: J Med Internet Res. 2023 Apr 19;25:e44035. doi: 10.2196/44035 (PMC10157465; doi:10.2196/44035)
Supplement: Multimedia Appendix 1 [file jmir_v25i1e44035_app1.docx]

**Appendix 1:**

**Key domains and associated search terms**

| **Key domains** | **Search terms for each domain** |
| --- | --- |
| eHealth | exp Technology/ OR exp Medical Records Systems, Computerized/ OR exp informatics/ OR exp computing methodologies/ OR  exp Technology, Radiologic/ OR exp Telecommunications/ OR exp Management Information Systems/ OR exp Diagnosis, Computer-Assisted/ OR ((communicat* or health* or informat* or comput* or medical) adj3 (technol* or system* or applicat* or process*)).tw,kf. OR(electronic adj3 record*).tw,kf. OR(ehealth or electronic health or telehealth or tele-health or telemedicine or tele-medicine or telecommunicat* or tele-communicat* or videoconferenc* or video-conferenc* or virtual care or teleradio* or tele-radio* or telemetry or mobile app* or informatics or computer-assist* or computer assist* or mobile health or mhealth or m-health or software or EHR? or EMR?).tw,kf. ((virtual or remote or distance or mobile or video) adj3 (consult* or health or medicine)).tw,kf. |
| Integrated care model | exp "Delivery of Health Care, Integrated"/ OR "Continuity of Patient Care"/ OR exp Patient Care Planning/ OR exp Patient-Centered Care/ OR  exp Patient Care Management/ OR Patient Care/ OR exp Interprofessional Relations/ OR ((Integrat* or multidisciplin* or interdisciplin* or interprofession* or team* or coordinat* or comprehensive or shar* or manage* or organi?ed or coop* or seamless or continu*) adj3 (care or healthcare or service* or deliver* or communicat* or relation* or treatment* or strateg* or program* or system*)).tw,kf. OR Team*.tw,kf. OR ((Case or cases or care or transition* or patient* or disease* or treatment*) adj3 (manage* or plan*)).tw,kf. OR(patient adj3 (cent?ed or tailored or integrat* or orient* or focus*) adj3 care).tw,kf. OR ((Linked or network* or structur*) adj3 care).tw,kf. OR (Care adj3 (coordinat* or continu* or guid* or transmural)).tw,kf. OR  ((Critical or clinical) adj2 pathway*).tw,kf. |
| Patients with complex care needs | exp Chronic Disease/ OR exp Comorbidity/ OR ((chronic* or complex or multi* or concurren* or co-occur* or co occur* or co-exist* or co exist* or dual or permanent or nonrevers* or non-revers*) adj2 (diagnos* or disease* or ill* or condition* or insufficienc* or disorder* or sick*)).tw,kf.OR (multimorbid* or multi-morbid* or comorbid* or co-morbid* or CCC).tw,kf. OR (poly-patholog* or polypatholog*).tw,kf. OR (pluri-patholog* or pluripatholog*).tw,kf. |
| Primary health care | (clinic* or practi* or primary or physician* or refer* or visit* or outpatient* or consult* or family or communit* or ambulatory or centre? or center? or office).ti,ab. |
