# Supplementary material for: Information and Communications Technologies Enabling Integrated Primary Care for Patients With Complex Care Needs: Scoping Review
Source: J Med Internet Res. 2023 Apr 19;25:e44035. doi: 10.2196/44035 (PMC10157465; doi:10.2196/44035)
Supplement: Multimedia Appendix 2 [file jmir_v25i1e44035_app2.docx]

**Appendix 2: Full search strategy for PsycInfo**

Database: PsycINFO

Search Strategy:

--------------------------------------------------------------------------------

1 exp integrated services/ (3363)

2 interdisciplinary treatment approach/ (7046)

3 multimodal treatment approach/ (1804)

4 adjunctive treatment/ (1118)

5 exp teams/ (15312)

6 "continuum of care"/ (1688)

7 ((Integrat* or interdisciplin* or inter-disciplin* or interprofession* or inter-profession* or team* or collab* or coordinat* or co-ordinat* or integrat* or comprehensive or shar* or manage* or organi?ed or coop* or seamless or continu*) adj3 (care or healthcare or service* or deliver* or communicat* or relation* or treatment* or strateg* or program* or system* or collab* or team* or approach* or model*)).tw,kw. (307619)

8 (multidisciplinary or multi-disciplinary or MDC).tw,kw. (19980)

9 (team* adj3 care*).tw,kw. (4698)

10 ((Case or cases or care or transition* or patient* or disease* or treatment*) adj3 (manage* or plan*)).tw. (56676)

11 (patient adj3 (cent?ed or tailored or integrat* or orient* or focus*) adj3 care).tw. (1029)

12 ((Linked or network* or structur*) adj3 care).tw. (2987)

13 (Care adj3 (coordinat* or continu* or guid* or transmural)).tw. (11754)

14 ((Critical or clinical) adj2 pathway*).tw. (734)

15 or/1-14 [integrated health care concept] (355701)

16 exp communication systems/ (36684)

17 exp electronic health services/ (8882)

18 exp electronic communication/ (28222)

19 exp computer applications/ (66652)

20 information systems/ (5296)

21 telemedicine/ (4768)

22 intelligent agents/ (975)

23 exp computer applications/ (66652)

24 exp information technology/ (156360)

25 exp communications media/ (76965)

26 exp electronic communication/ (28222)

27 teleconferencing/ (863)

28 telemedicine/ (4768)

29 telecommunications media/ (1377)

30 telemetry/ (153)

31 online therapy/ (2640)

32 exp mobile devices/ (7012)

33 exp computers/ (40062)

34 exp technology/ (203691)

35 ((communicat* or health* or informat* or comput* or medical) adj3 (technol* or system* or applicat* or process*)).tw. (133589)

36 ((informat* or communicat*) adj3 (exchang* or tech*)).tw,kw. (23542)

37 (electronic adj3 record*).tw. (3865)

38 (ehealth or e-health or electronic health or telehealth or tele-health or telemedicine or tele-medicine or telecommunicat* or tele-communicat* or videoconferenc* or video-conferenc* or virtual care or teleradio* or tele-radio* or telemetry or mobile app* or informatics or mobile health or mhealth or m-health or software or EHR? or EMR?).tw,kw. (38028)

39 ((virtual or remote or distance or mobile or video) adj3 (consult* or health or medicine)).tw. (1882)

40 or/16-39 [eHealth Technology concept] (381226)

41 "chronicity (disorders)"/ (4348)

42 exp sequelae/ (19348)

43 exp chronic illness/ (27561)

44 comorbidity/ (30314)

45 dual diagnosis/ (2010)

46 ((chronic* or complex or multi* or concurren* or co-occur* or co occur* or co-exist* or co exist* or dual or permanent or nonrevers* or non-revers*) adj2 (diagnos* or disease* or ill* or condition* or insufficienc* or disorder* or sick*)).tw,kw. (67756)

47 (complex adj3 patient*).tw,kw. (2358)

48 (multimorbid* or multi-morbid* or comorbid* or co-morbid* or CCC).tw. (57545)

49 (poly-patholog* or polypatholog*).tw. (30)

50 (pluri-patholog* or pluripatholog*).tw. (4)

51 or/41-50 [Chronic illness concept] (160385)

52 15 and 40 and 51 [Integrated care concept + eHealth Technology concept + Chronic Illness concept] (3116)

53 limit 52 to yr="2000 -Current" (2896)

***************************
